# Inhibition of miR-20a promotes neural stem cell survival under oxidative stress conditions

**DOI:** 10.3389/fnins.2025.1601101

**Published:** 2025-06-26

**Authors:** Ivan Arzhanov, Ruslan A. Klassen, Lukas Valihrach, Nataliya Romanyuk

**Affiliations:** ^1^Department of Neuroregeneration, Institute of Experimental Medicine CAS, Prague, Czechia; ^2^Department of Neuroscience, 2nd Medical Faculty, Charles University, Prague, Czechia; ^3^Laboratory of Glial Biology and Omics Technologies, Institute of Biotechnology of the Czech Academy of Sciences – BIOCEV, Vestec, Czechia; ^4^Department of Biochemistry and Microbiology, Faculty of Food and Biochemical Technology, UCT Prague, Prague, Czechia

**Keywords:** microRNA-20a, neural stem cells, oxidative stress, neural apoptosis, neuroprotection

## Abstract

**Introduction:**

Oxidative stress (OS) is a key contributor to secondary damage following spinal cord injury (SCI), leading to neural stem cell (NSC) dysfunction and apoptosis. MicroRNA-20a (miR-20a) is upregulated after SCI and plays a role in regulating apoptosis and survival pathways. This study explores the therapeutic potential of miR-20a inhibition in mitigating OS-induced damage in NSCs.

**Methods:**

Human iPSC-derived NSCs were subjected to oxidative stress by exposure to 100 µM hydrogen peroxide (H_2_O_2_) for 2 hours, followed by treatment with a miR-20a inhibitor (100 nM) to attenuate the adverse effects. Metabolic activity was evaluated using the Alamar Blue assay. Apoptotic responses and miR-20a expression levels were assessed via flow cytometry, RT-qPCR, and Western blot analysis.

**Results:**

NSCs exposed to OS showed a marked reduction in metabolic activity. However, treatment with a miR-20a inhibitor over 72 h significantly improved cell survival and metabolic activity in a time-dependent manner compared to untreated stressed cells.

**Discussion:**

Our findings suggest that miR-20a inhibition mitigates OS-induced cytotoxicity and promotes NSC viability, presenting a potential therapeutic approach for enhancing neural tissue regeneration.

## 1 Introduction

The pathophysiology of spinal cord injury (SCI) is a complex and multifunctional process affecting the nervous, vascular, and immune systems. Pathology progresses within months of the initial injury, making SCI a challenging condition to understand and effectively treat (Strickland et al., 2011). The pathophysiology of spinal cord injury can be conditionally divided into two distinct phases (Uyeda and Muramatsu, 2020).

The primary injury phase begins with an initial mechanical injury, which may manifest as contusion or compression (Anjum et al., 2020). The secondary phase, emerging within minutes of the primary injury, may persist for weeks to months. It is accompanied by dysregulation of the NMDA, AMPA, and kainate receptors, leading to a disturbance in glutamate balance, thereby promoting excitotoxicity within the surrounding uninjured tissue. Ionic imbalances lead to the phosphorylation of mitochondria and increase Ca^2+^ influx, signifying the initiation of the subacute phase. This phase is further marked by heightened levels of reactive oxygen species (ROS) and lipid peroxidation, thereby actively contributing to neuroinflammation (Dimitrijevic et al., 2015).

Reactive oxygen species and reactive nitrogen species (RNS) produced as a consequence of damage induce oxidative stress (OS) on healthy neurons, affecting their proteins, lipids, and DNA, thereby expediting the progression of neurodegeneration (Wildburger et al., 2009). ROS, including hydrogen peroxide (H_2_O_2_), superoxide (O_2_^−^), and hydroxyl radical (HO^−^), contribute to increased permeability of the blood–brain barrier, modifications to tubulin, and disruptions in synaptic transmission (Stohs and Bagchi, 1995). Furthermore, ROS-induced activation of associated signaling pathways can lead to neuronal apoptosis or neurodegeneration (Galluzzi et al., 2009; Liang et al., 2010).

Two major apoptotic pathways, the death receptor-initiated (extrinsic) pathway, and the mitochondrial (intrinsic) pathway, are induced through caspase activation (Liang et al., 2010). The Bcl family proteins, including pro-apoptotic (Bax and Bak) and anti-apoptotic (Bcl-2 and Bcl-XL) proteins, maintain a delicate balance under normal conditions (Galluzzi et al., 2009). Injury disrupts this balance, leading to the generation of BH3-interacting-domain death agonist (Bid) proteins. In apoptosis, phospholipid membrane asymmetry shifts, with phosphatidylserine (PS) appearing on the outer leaflet, facilitating recognition and removal through phagocytosis. Extracellular annexin V can influence membrane particle separation, potentially delaying the activation of the internal cascade during intracellular proapoptotic events (Connor et al., 1992; Devaux, 1991).

In addition to ROS, the production of pro-inflammatory cytokines in the extracellular space during SCI can induce apoptosis and necrosis, even in otherwise healthy tissue. Under normal conditions, programmed cell death serves as a crucial mechanism for preventing the spread of lesions and eliminating damaged cells (Alizadeh et al., 2019). However, in the context of the central nervous system, marked by its lack of regenerative capacity, this process can lead to irreversible and profound consequences.

One of the key mechanisms that regulates neuroinflammation and neuronal apoptosis during SCI is the JAK2/STAT3 signaling pathway. In various scenarios, STAT3 plays a central role in cell survival. For example, it promotes B cell proliferation by inhibiting apoptosis, achieved through the induction of the antiapoptotic gene Bcl-2 (Alonzi et al., 2001). Furthermore, STAT3 activation is required for interleukin-10 to exhibit its anti-inflammatory properties on macrophages (Riley et al., 1999). In these contexts, STAT3 primarily functions to ensure cell survival, facilitating the induction of antiapoptotic genes like Bcl-2 and Bcl-XL (Alonzi et al., 2001). Interestingly, in some cases, Bcl-2 is induced even in the absence of STAT3, contrary to the expected pro-survival role of STAT3 (Estaquier et al., 2012).

Another important response mechanism to SCI is the activation of the mTOR pathway (Maiese, 2015). In the acute stage of SCI, mTOR is crucial for controlling processes that lead to subsequent injuries, such as inflammation, cell death, and macrophage/microglia activation (Sekiguchi et al., 2012). Inhibition of mTOR has been shown to reduce cell death in damaged neural tissue, decrease levels of pro-inflammatory markers, and suppress the activity of nitric oxide synthase (NOS) induced by cytokines in microglia (Dello Russo et al., 2009). Moreover, inhibiting the mTOR signaling pathway has been suggested to decrease inducible NOS expression and microglial activation, thereby mitigating neuronal injury (Lu et al., 2006). Interestingly, mTOR activation often acts as a protective mechanism against apoptotic cell death in the nervous system (Tang et al., 2014). Conversely, a reduction in mTOR activity contributes to neuronal apoptosis and exacerbates OS pathways (Chen et al., 2010).

It is known that essential cellular processes can be regulated by microRNAs. MicroRNAs (miRNAs), small non-coding RNA molecules, exert their influence by suppressing gene expression through mechanisms like inhibiting protein synthesis and promoting mRNA degradation (Martinez and Peplow, 2020). MiRNAs, with their ability to interact with multiple mRNA molecules through partial complementarity, play pivotal and multifunctional roles within the central nervous system (CNS), significantly contributing to various fundamental processes. MiRNAs are well-established key players in the pathogenesis of SCI, controlling cellular state and functionality through complex post-transcriptional silencing mechanisms affecting numerous genes (Nieto-Diaz et al., 2014).

MiR-20a is firmly established as a critical contributor to the pathophysiology of SCI (Jee et al., 2012). Its upregulated expression persists for at least 1 week after SCI, indicating its crucial role in preventing neural tissue regeneration (Liu et al., 2009; Yunta et al., 2012). Interestingly, antisense miR-20a infusion yields remarkable results, reactivating pivotal signaling pathways such as STAT3/Jak2/ERK1/2 and enhancing PI3K/Akt phosphorylation. Concurrently, it downregulates apoptotic signals, reducing key pro-apoptotic factors like Bax and cytochrome c. MiR-20a’s impact extends directly targeting STAT3, a central mediator in the SCI response, emphasizing its multifaceted influence on SCI pathways (Carraro et al., 2009). Furthermore, miR-20a’s role in regulating apoptotic pathways after SCI is underscored by its impact on myeloid cell leukemia protein sequence-1 (Mcl-1) (Liu et al., 2015).

In our study, we examined in more detail the aspects of oxidative stress and its impact on neural apoptosis and microRNA-mediated regulation. Understanding these processes is essential in the search for innovative therapeutic strategies to alleviate the effects of SCI and improve the recovery prospects of affected individuals.

## 2 Materials and methods

### 2.1 Human induced pluripotent stem cell-derived neural precursors

The human induced pluripotent stem cell (hiPSC) line used in this study was derived from female fetal lung fibroblasts (IMR90) obtained from ATCC (Manassas, VA, United States). The cells were transduced using a lentivirus-mediated delivery of human cDNAs for OCT4, SOX2, NANOG, and LIN28, as previously described (Romanyuk et al., 2015). Details on clone selection, hiPSC line validation, and neural progenitor (NP) derivation are available in Polentes et al. (2012).

Briefly, early NPs were generated under low-attachment culture conditions in the presence of Noggin (500 ng/mL, R&D Systems, Minneapolis, MN, United States), SB 431542 (10 nM, a TGF-*β* pathway inhibitor, Sigma-Aldrich, St. Louis, MO, USA), basic fibroblast growth factor (bFGF, 10 μg/mL), and brain-derived neurotrophic factor (BDNF, 20 μg/mL), [both sourced from PeproTech (London, UK)]. To confirm that the derived cells possess neural precursor properties, we performed immunostaining for several specific markers. The results are presented in Supplementary Figure 1. The hiPSC-derived NPs were maintained in tissue culture flasks coated with poly-L-ornithine (0.002% in distilled water) and laminin (10 μg/mL in DMEM), both from Sigma-Aldrich. The growth medium consisted of a 1:1 mixture of DMEM and Neurobasal medium, supplemented with B27 (1:50) and N2 (1:100) (Gibco, Life Technologies, Grand Island, NY, United States), penicillin–streptomycin (50 U/mL, Gibco), FGF (10 ng/mL), EGF (10 ng/mL), and BDNF (20 ng/mL) (PeproTech). Media were replaced three times per week.

### 2.2 Experimental design

The experimental workflow consisted of three key components:

#### 2.2.1 Flow cytometry analysis

Flow cytometry was used to evaluate the effects of various H_2_O_2_ concentrations under OS conditions. This provided insight into the optimal concentration required to induce apoptosis while minimizing excessive cytotoxicity.

#### 2.2.2 RT-qPCR analysis

RT-qPCR was performed to determine the optimal period of time for miR-20a downregulation. This analysis was essential for understanding the functional role of miR-20a inhibition in cellular stress responses.

#### 2.2.3 Western blot analysis

Western blotting was used to assess changes in protein expression levels following miR-20a inhibition (miRCURY LNA™ miRNA Inhibitor EXIQON #3392203) under OS conditions. Cell cultures were treated with 100 nM miR-20a inhibitor in the culture medium for 72 h. The optimal working concentration of the inhibitor was determined based on Western blot and immunostaining analyses (data not shown). A scrambled miRNA inhibitor (Negative Control, EXIQON #199007–121), with no predicted targets in the human genome, was used as a negative control.

#### 2.2.4 Metabolic activity measurements

Cellular metabolic activity was evaluated at 2, 24, 48, and 72 h after beginning of experiment (indicated by the pink arrow) using appropriate assays to monitor cellular recovery and stress responses over time.

**Figure 1:**
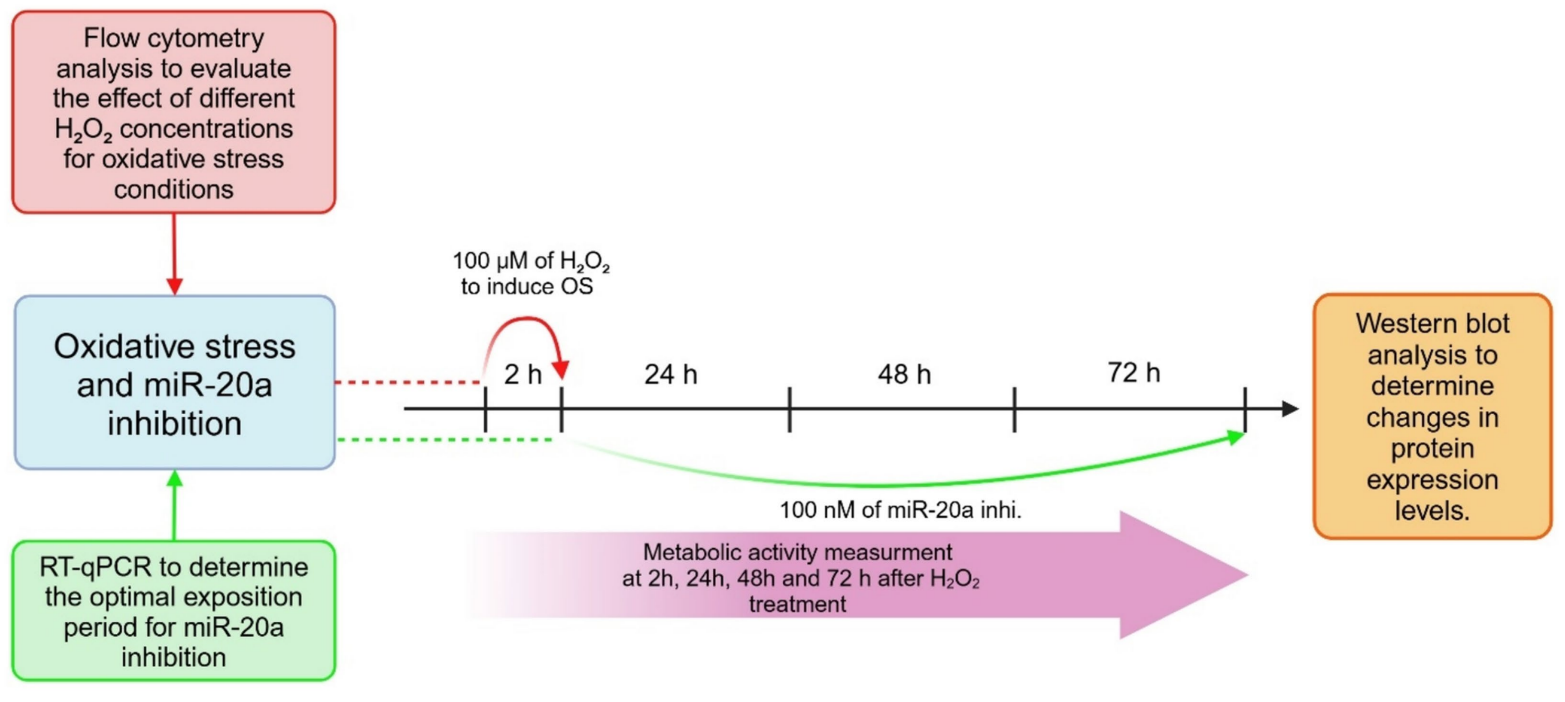
Experimental design. The figure outlines the experimental design used to investigate the effects of oxidative stress and miR-20a inhibition on cellular apoptotic processes.

The experimental timeline highlights critical points for interventions and measurements, ensuring a comprehensive evaluation of the effects of OS and miR-20a inhibition on neural stem cells (Figure 1).

### 2.3 Oxidative stress

Oxidative stress was induced by treating cell cultures with 100 μM hydrogen peroxide (H_2_O_2_). To prepare the working solution, 2 μL of H_2_O_2_ solution (30% w/w in H_2_O; H1009, Sigma-Aldrich, Spain) was diluted in 880 μL of distilled water (dH_2_O) receiving 20 mM stock solution, and then further diluted 1: 200 in NSC culture medium to achieve working concentration of 100 μM.

Cells were exposed to 100 μM H_2_O_2_ for 2 h to induce OS, after which the medium was replaced with fresh NSC culture medium to allow recovery under normal conditions.

### 2.4 Flow cytometry

After treatment with different concentrations of H_2_O_2_ (100, 200, and 500 μM), cells were collected by enzymatic treatment and centrifugation (5 min, 900 × g, room temperature). Approximately 2 × 10^5^ cells were resuspended in 50 μL of Annexin V Binding Buffer (ABB) with Annexin V Dyomics 647 (both from Exbio) at a concentration of 1 μL/100 μL ABB. Additionally, several control samples of untreated cells were prepared: (i) unstained cells in ABB, (ii) single-stained cells with Annexin V (ABB + A), (iii) single-stained cells with Hoechst 33258 (Sigma-Aldrich) (ABB + H), and (iv) double-stained cells (ABB + A + H). All samples were incubated on ice in a dark environment for 30 min, centrifuged, and resuspended in 150 μL of ABB. Hoechst 33258, at a final concentration of 1 μg/mL, was added 5 min before measurement. Measurements were performed on a BD LSRFortessa™ SORP flow cytometer. The filters used were as follows: Annexin V (APC) – excitation laser at 640 nm (40 mW) with an emission filter of 670/14 nm; Hoechst 33258 – excitation laser at 405 nm (50 mW) with an emission filter of 450/50 nm. After initial gating for cell size and shape using SSC-A × FSC-A and excluding doublets through FSC-H × FSC-A, a donor versus acceptor channel plot was used to identify the population positive for both fluorophores (P1). Hoechst 33258-positive cells were detected using the Pacific Blue channel with a gain of 357 V, while Annexin V (APC) was detected using the APC channel with a gain of 451 V. The P1 population was then projected as a histogram of the FRET channel-to-donor channel ratio (FRET ratio) to establish gates for FRET-positive and FRET-negative sorting.

### 2.5 Reverse transcription and quantitative PCR

RNA was isolated using the standard TRIzol protocol (Sigma). Reverse transcription (RT) reactions were conducted using the qScript Flex cDNA Kit (Quantabio) in a total reaction volume of 10 μL. Each reaction included 2 μL of template RNA, 1 × buffer, 0.05 μM of Two-tailed RT primers, 1 μL of GSP enhancer, 0.5 μL of RT enzyme, and nuclease-free water to reach the final volume. The reactions were incubated in a CFX 1000 thermocycler (Bio-Rad) under the following conditions: 45 min at 25°C, followed by 5 min at 85°C, and then held at 4°C. After incubation, cDNA was diluted by adding 50 μL of nuclease-free water.

Quantitative PCR (qPCR) was performed in a 10 μL reaction volume. Each reaction contained 1 × SYBR Grandmaster Mix (TATAA Biocenter), forward and reverse primers (final concentration of 0.4 μM each), and 2 μL of the diluted cDNA template (resulting in a final cDNA dilution of 15×). The qPCR reactions were run in duplicate using a 384-well plate on a CFX 384 Real-Time Detection System (Bio-Rad). The cycling protocol included an initial denaturation step at 95°C for 30 s, followed by 45 cycles of 95°C for 5 s and 60°C for 15 s. A melting curve analysis was performed at the end of each run to confirm the specificity of amplification.

### 2.6 Metabolic activity (Alamar Blue test)

Resazurin sodium, also known as Alamar Blue (7-Hydroxy-3H-phenoxazin-3-one-10-oxide sodium salt) (Sigma-Aldrich, St. Louis, MO, United States), was used to assess the metabolic activity of NSCs following hydrogen peroxide treatment and miR-20a inhibitor (Qiagen, Hilden, Germany) application. A 10% Alamar Blue (AB) solution was added to the NSCs, and the cells were incubated for 3 h. After the incubation period, the supernatant was collected for further measurement.

The fluorescence intensity of the reduced AB was measured using a TECAN GENios microplate reader (Tecan, Männedorf, Switzerland) with an excitation wavelength of 550 nm and an emission wavelength of 590 nm. The metabolic activity was calculated as the ratio of fluorescence in experimental samples to that of blank samples, which consisted of the same medium without cells. Results were expressed in relative fluorescence units (RFU). To standardize the data, the fluorescence values of treated samples were normalized to the corresponding control samples at each time point.

### 2.7 Western blotting

Cells were homogenized on ice using RIPA lysis buffer (Sigma-Aldrich), supplemented with a protease and phosphatase inhibitor cocktail and EDTA (Thermo Scientific). After a 40-min incubation at 4°C, the homogenate was centrifuged at 14,000 RPM for 20 min (Eppendorf 5,804 R centrifuge), and the supernatant was collected and stored at −20°C. Protein concentration was determined using the Pierce™ BCA Protein Assay Kit (Thermo Scientific) and quantified with i-control software on a Tecan Infinite^®^ 200 PRO Multimode Reader. Samples for WB analysis were mixed with SDS sample buffer (80 mM Tris, pH 6.8; 2% SDS; 10% glycerol; 0.0006% bromophenol blue; 0.1 M DTT; all from Sigma-Aldrich) in a ratio of 1:1 and denatured at 95°C for 5 min. SDS-PAGE was performed using Bio-Rad Mini-PROTEAN TGX™ Precast Gels (8–14% gradient) in running buffer (25 mM Tris, 192 mM glycine, 0.1% SDS; Sigma-Aldrich). A total of 40 μg of protein per sample was loaded into each well. Proteins were separated by electrophoresis for 10 min at 30 mA and 35 min at 60 mA and then transferred onto PVDF membranes (Life Technologies) using transfer buffer (25 mM Tris, 192 mM glycine, 20% methanol, pH 8.3) for 1 h at 350 mA. The proteins on the membranes were visualized with Ponceau S Staining Solution (Cell Signaling Technology) and washed with Tris-buffered saline containing Tween-20 (TBST; 20 mM Tris, 150 mM NaCl, 0.1% Tween-20, pH 7.5). Blocking was performed with a 5% milk solution (Cell Signaling Technology) for 1 h at room temperature.

For immunodetection, membranes were incubated overnight at 4°C with primary antibodies diluted 1:1,000 in either a 5% bovine serum albumin (BSA) solution, a 5% Nonfat dry milk solution (both are from Cell Signaling Technology, Danvers, Massachusetts, USA), or TBST, depending on the antibody. Membranes were then washed with TBST and incubated for 1 h at room temperature with secondary antibodies diluted in TBST. After further washes, protein detection was performed using the SuperSignal™ West Dura Chemiluminescent Substrate (Thermo Scientific) according to the manufacturer’s protocol.

Several primary antibodies were used to detect the proteins of interest, as listed in Table 1. All primary antibodies were sourced from Cell Signaling Technology (Danvers, Massachusetts, USA), except for *β*-Actin (45 kDa), which was obtained from Sigma-Aldrich and used as an endogenous control.

**Table 1:** The list of the primary antibodies.

Antibodies name	Catalog number #	Molecular weight (kDa)	Isotope	Dilution solution	Dilution
mTOR	2983	289	Rabbit mAb	BSA	1:1,000
Akt	9272	60	Rabbit mAb	BSA	1:1,000
PTEN	9559	54	Rabbit mAb	BSA	1:1,000
Phospho-Akt	4060	60	Rabbit mAb	BSA	1:1,000
Stat3	9139	79, 86	Mouse mAb	Milk	1:1,000
Pospho- Stat3	9145	79, 86	Rabbit mAb	BSA	1:2,000
PARP	9542	116	Rabbit mAb	BSA	1:1,000
Cleaved PARP	9541	89	Rabbit mAb	BSA	1:1,000
Mcl-1	94296	40	Rabbit mAb	BSA	1:1,000
Bcl-XL	2764	30	Rabbit mAb	BSA	1:1,000
Bax	14796	20	Rabbit mAb	BSA	1:1,000
Caspase-3	14220	35, 19, 17	Rabbit mAb	BSA	1:1,000
Cytochrome c	11940	14	Rabbit mAb	BSA	1:1,000
β-Actin	A2228	42	Mouse mAb	TBST	1:2,000

For the detection of primary antibodies, peroxidase-conjugated AffiniPure Goat Anti-Rabbit (#164225) and Anti-Mouse IgG (#131224) (H + L) (Jackson ImmunoResearch Laboratories inc., Baltimore Pike, USA) secondary antibodies were used.

Blots were visualized using an Azure c600 Imaging System (Azure Biosystems, Dublin, CA 94568 USA) and analyzed with Fiji software. Band intensities were normalized to β-Actin as an endogenous control for quantitative comparisons. Western blot data were normalized to time-matched controls, with the control group set to 1. The results are presented as fold changes in treated samples relative to their respective controls. Data are expressed as the mean ± SEM, based on at least three independent experiments.

### 2.8 Statistical analysis

Statistical differences between groups were assessed using repeated one-way ANOVA. Analyses and graphical representations were performed using Prism 8.0.2 software (GraphPad Software Inc.). Differences were considered statistically significant when the *p*-value was less than 0.05 (*p* < 0.05).

## 3 Results

### 3.1 Oxidative stress conditions

To investigate apoptotic processes in NSCs, we employed an OS *in vitro* model. To find an optimal condition for creating the conditions of oxidative stress and provoking the beginning of apoptotic processes in the cells, we tested several concentrations of H_2_O_2_: 100, 200, and 500 μM. After a 2-h treatment with various concentrations of H_2_O_2_, the cells were collected and stained with Annexin V, which binds to phosphatidylserine and serves as a marker for early apoptosis, and Hoechst, a DNA-binding dye used to indicate later stages of apoptosis. In our experiment, the Pacific Blue channel was set to a gain of 357 V. This setting allowed us to distinguish Hoechst-positive cells, characterized by high signal intensity due to chromatin condensation in apoptotic cells, from cells with dimmer staining, indicative of normal chromatin. All together we organized groups: Control (untreated cells), cells stained only with Annexin V, cells stained with Hoechst, cells which were treated with 100 μM, 200 μM, and 500 μM of H_2_O_2_ and stained with Annexin V and Hoechst. Cell analysis was performed using flow cytometry, and the results were expressed as the percentage of cells of various categories: Annexin V-positive cells (early apoptotic), Hoechst-positive cells (late apoptotic), cell fragments (suggesting complete cell disintegration), and alive cells. The scatter plots from the flow cytometry analysis of cells are presented in Supplementary Figure 2.

**Figure 2:**
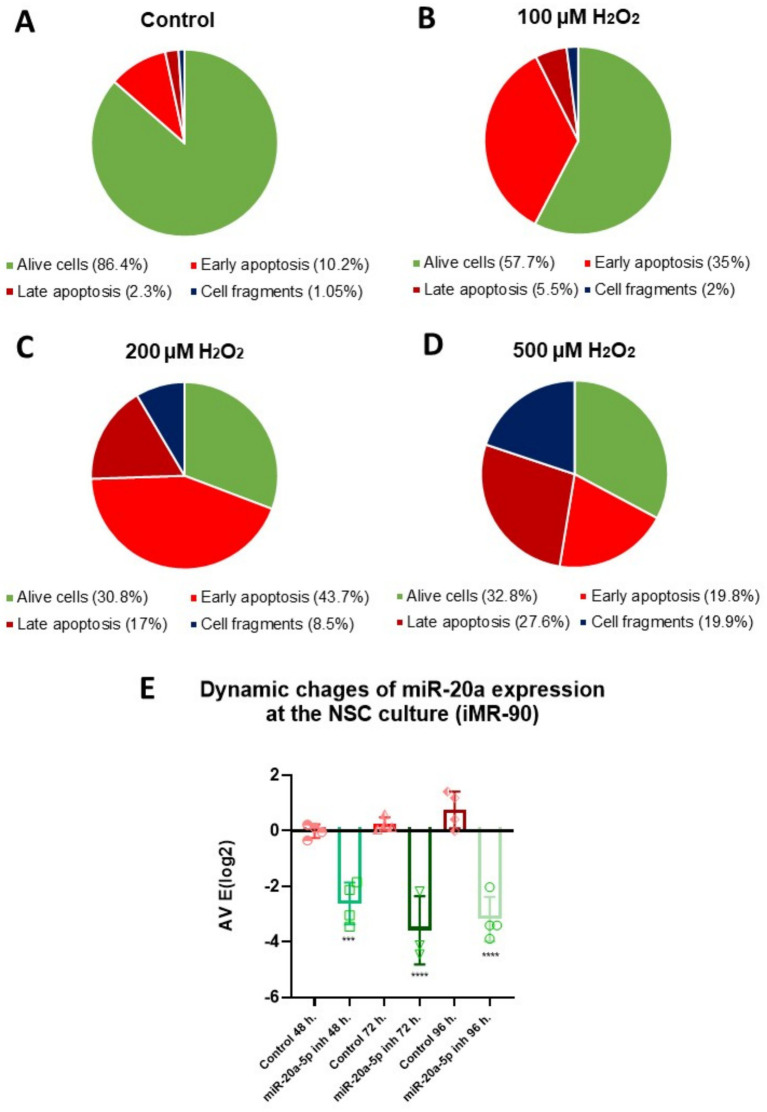
Optimization of H_2_O_2_ concentration and period of exposition for miR-20a inhibition in NSCs: apoptosis analysis via flow cytometry and qPCR. **(A–D)** Flow cytometry analysis showing the distribution of cell populations (Alive cells, Early Apoptosis, Late Apoptosis, and Cell Fragments) in NSCs treated with varying concentrations of hydrogen peroxide (H_2_O_2_). Pie chart depicts cell percentages under different conditions: **(A)** Control (untreated cells), **(B)** 100 μM H_2_O_2_, **(C)** 200 μM H_2_O_2_, and **(D)** 500 μM H_2_O_2_. Early apoptotic cells were identified with Annexin V staining, while Hoechst staining detected cells in the late apoptotic stage. Panel E: Quantitative PCR (qPCR) results show a time-dependent inhibition of miR-20a expression, with the most significant reduction at 72 h post-treatment. Statistical significance was evaluated using One-way ANOVA (***p < 0.001, ****p < 0.0001; *F* (5, 17) = 28.39).

The results showed that 200 μM and 500 μM concentrations of H_2_O_2_ had a strong cytotoxic effect, leading to cell death. A high percentage of cells were in the late stages of apoptosis (17 and 27.6%, respectively) or already dead (8.5 and 19.9%, respectively) (Figures 2A–D). In contrast, the 100 μM concentration of H_2_O_2_ induced apoptosis without causing immediate cell death. In this group, 35% of the cells were Annexin V-positive, indicating early apoptotic activity. The results of the flow cytometry analysis, depicting the distribution of cells based on positive staining for Annexin V, Hoechst, and the combination of Annexin V and Hoechst, are shown in Supplementary Figure 1. Given that apoptosis in the later stages is generally irreversible, but the process can potentially be halted or slowed during the early stages, we chose to use 100 μM hydrogen peroxide as our model for OS that induces apoptosis in cells.

Our next objective was to determine the optimal period of exposition for downregulating miR-20a. To achieve this, we incubated NSCs with 100 nM of a miR-20a inhibitor and measured their expression at 48, 72, and 96 h using qPCR (Figure 2E). The results showed a gradual decrease in miR-20a expression from 48 to 96 h (*p* < 0.001 and *p* < 0.0001; *F* (5, 17) = 28.39), with the lowest expression observed at 72 h (*p* < 0.0001; F (5, 17) = 28.39) of incubation with the inhibitor.

Our results show that 100 μM H_2_O_2_ effectively induces early apoptosis in NSCs without immediate cell death, making it an optimal concentration for modeling oxidative stress. Additionally, treatment with a miR-20a inhibitor caused the greatest downregulation of miR-20a expression at 72 h, identifying this as the ideal time point for studying its role in OS.

### 3.2 Inhibition of miR-20a partly recovers cell metabolic activity altered by oxidative stress

The effects of OS and miR-20a inhibition on cell metabolic activity were assessed using Alamar Blue assays. Four experimental groups were created: (i) Control (untreated cells), (ii) miR-20a inhibitor-treated cells, (iii) H_2_O_2_ and miR-20a inhibitor-treated cells, and (iv) H_2_O_2_ and normal medium (without inhibitor) treated cells. Measurements were taken at multiple time points: 2 h post-oxidative stress induction for the Control and H_2_O_2_ groups, and 24, 48, and 72 h for all groups.

Two hours after H_2_O_2_ treatment, measurements were conducted to compare the Control and H_2_O_2_-treated groups to determine if OS affects cell metabolism in the early stages following H_2_O_2_ application. Measurements in the remaining groups were not necessary, as qPCR results indicated that more time is required for miR-20a inhibition to exert its effects on the cells. No significant differences in cell metabolic activity were observed between the Control and H_2_O_2_-treated groups. These results suggest that the effect of ROS produced as a result of OS on the metabolic activity of NSCs does not manifest immediately.

**Figure 3:**
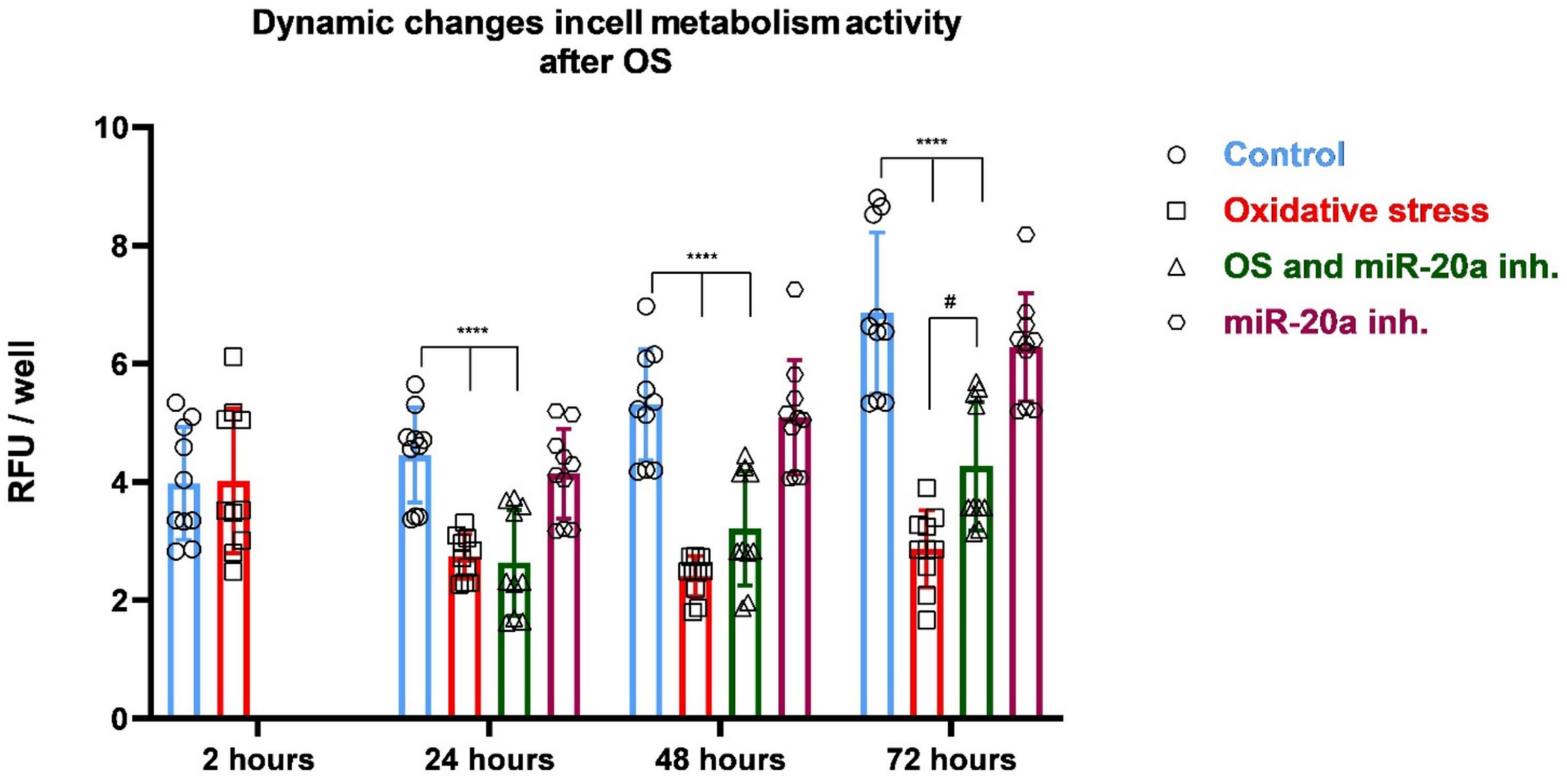
Changes of cell metabolic activity in response to oxidative stress and miR-20a inhibition. The figure presents dynamic changes in cell metabolic activity under oxidative stress and miR-20a inhibition. Metabolic activity was measured in four groups: Control (untreated), Oxidative Stress (H_2_O_2_-treated), miR-20a inhibitor-treated, and H_2_O_2_ together with miR-20a inhibitor-treated cells. Statistical significance was evaluated using One-way ANOVA #*p* < 0.05 and ****p < 0.0001 (*F* (13, 126) = 22.04). The results indicate that miR-20a inhibition partially restored metabolic activity in NSCs following OS.

Twenty-four hours after beginning of experiment, cells in the Control group maintained a high level of metabolic activity, while the H_2_O_2_-treated group continued to show suppressed metabolic function (*p* < 0.001 compared to the Control; *F* (13, 126) = 22.04) (Figure 3). The H_2_O_2_ and miR-20a inhibitor group showed significantly higher activity than the H_2_O_2_-only group (*p* < 0.05; F (13, 126) = 22.04), suggesting an early protective or reparative effect of miR-20a inhibition. At 48 h, the metabolic activity of the cells in the Control group remained stable, showing consistent metabolic activity. The miR-20a inhibitor-treated group (without H_2_O_2_) also maintained similar levels to the Control group, confirming the non-toxic nature of the miR-20a inhibitor under normal conditions. In contrast, the H_2_O_2_-treated groups demonstrate a gradual decrease in metabolic activity with time. The group of cells treated with H_2_O_2_ and miR-20a inhibitor showed a further increase in metabolic activity compared to 24 h after OS initiation, approaching levels similar to the level of Control groups (*p* < 0.001; F (13, 126) = 22.04). The H₂O₂-only group, however, remained significantly impaired. By 72 h, a clear trend of decline in metabolic activity was observed. These findings indicate that oxidative stress gradually impairs the metabolic activity of neural stem cells over time, rather than causing immediate damage. Notably, miR-20a inhibition enhances cellular recovery under oxidative conditions without affecting baseline metabolism, highlighting its potential protective role.

### 3.3 Changes in pro- and anti-apoptotic proteins expression under oxidative stress conditions

Pro-apoptotic proteins, such as Bax, Bad, and caspase-3, promote mitochondrial dysfunction and cytochrome c release, leading to the activation of the caspase cascade and neural cell death. In contrast, anti-apoptotic proteins, including Bcl-2, Bcl-XL, and Mcl-1, act to preserve mitochondrial integrity and inhibit the apoptotic machinery, offering potential neuroprotective effects. Following SCI, the dysregulation of these proteins tips the balance toward apoptosis, exacerbating neural loss and functional impairment.

**Figure 4:**
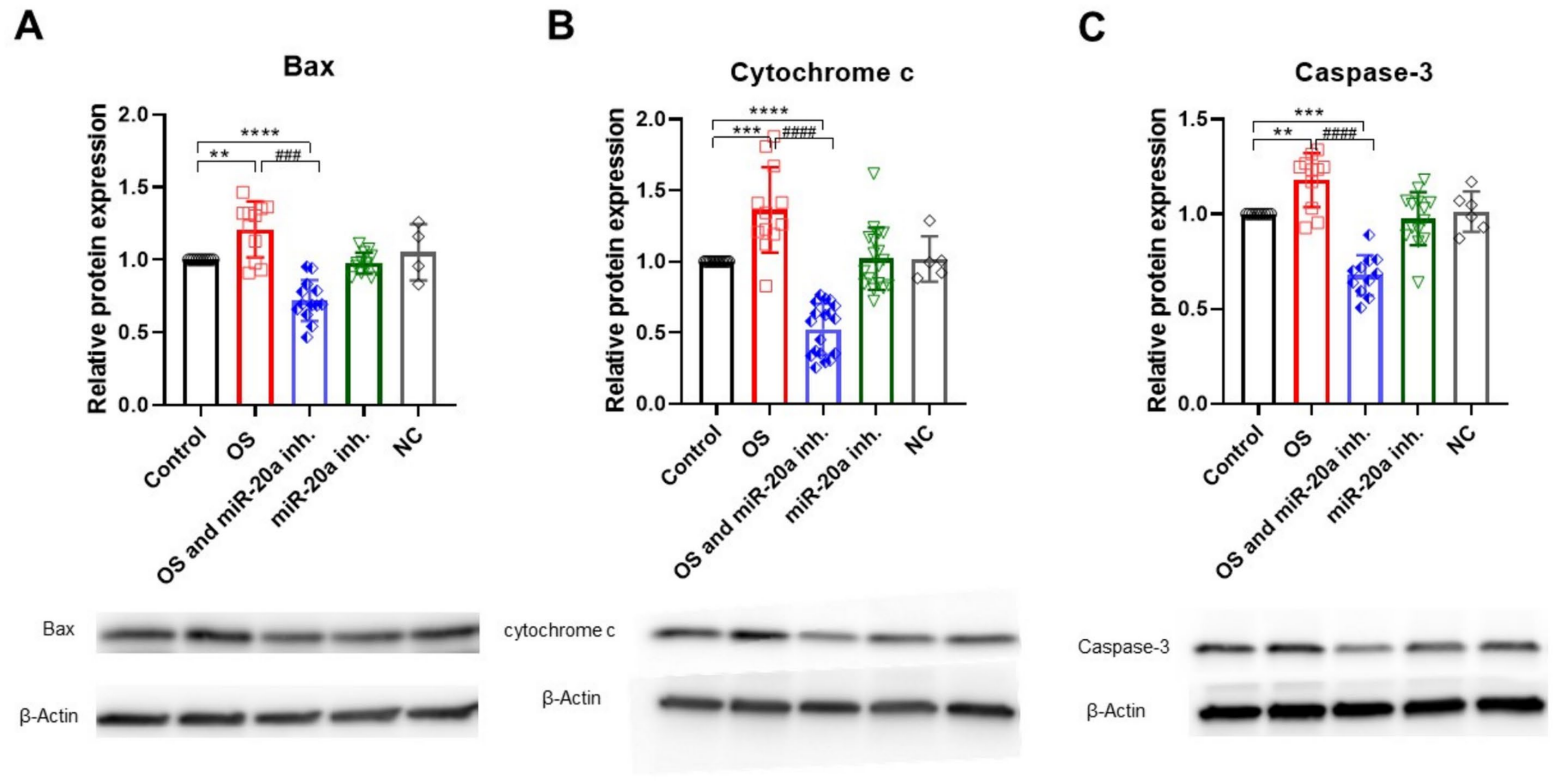
Pro-apoptotic protein expression under OS and miR-20a inhibition. Western blot analysis of pro-apoptotic proteins—Bax **(A)**, Cytochrome c **(B)**, and Caspase-3 **(C)**—under oxidative stress (H_2_O_2_) and miR-20a inhibition conditions. Protein levels were normalized to *β*-Actin and expressed relative to the control group (Control = 1). Bax **(A)**: H_2_O_2_ treatment significantly upregulated Bax expression (**p < 0.01), while miR-20a inhibition reduced Bax levels (###*p* < 0.001). Cytochrome c **(B)**: H_2_O_2_ caused a substantial increase in Cytochrome c (***p < 0.001), which was reduced by miR-20a inhibition (###*p* < 0.001). Caspase-3 **(C)**: Caspase-3 levels increased after H_2_O_2_ treatment (**p < 0.01) but decreased significantly with miR-20a inhibition (***p < 0.001). No significant changes were observed in the NC (negative control), highlighting the specific protective effect of miR-20a inhibition. Statistical significance was evaluated using One-way ANOVA (**p < 0.01, ***p < 0.001****p < 0.0001); Bax (*F* (4, 46) = 21.74); Cytochrome c (*F* (4, 61) = 33.70); Caspase-3 (*FF* (4, 46) = 26.38).

Bax is a crucial pro-apoptotic member of the Bcl-2 family that facilitates the release of cytochrome c from mitochondria, leading to the activation of caspase-9 and ultimately caspase-3, which drives the execution phase of apoptosis. Western blot analysis shows that Bax and cytochrome c levels significantly increase in groups treated with H_2_O_2_ (*p* < 0.01 for Bax (*F* (4, 46) = 21.74)) and *p* < 0.001 (*F* (4, 61) = 33.70) for cytochrome c (Figures 4A,B). However, the inhibition of miR-20a for 72 h notably reduces Bax and cytochrome c levels (*p* < 0.0001 for Bax (F (4, 46) = 21.74) and *p* < 0.0001(F (4, 61) = 33.70) for cytochrome c). In the group treated only with miR-20a inhibitor, no significant changes were observed compared to the control.

**Figure 5:**
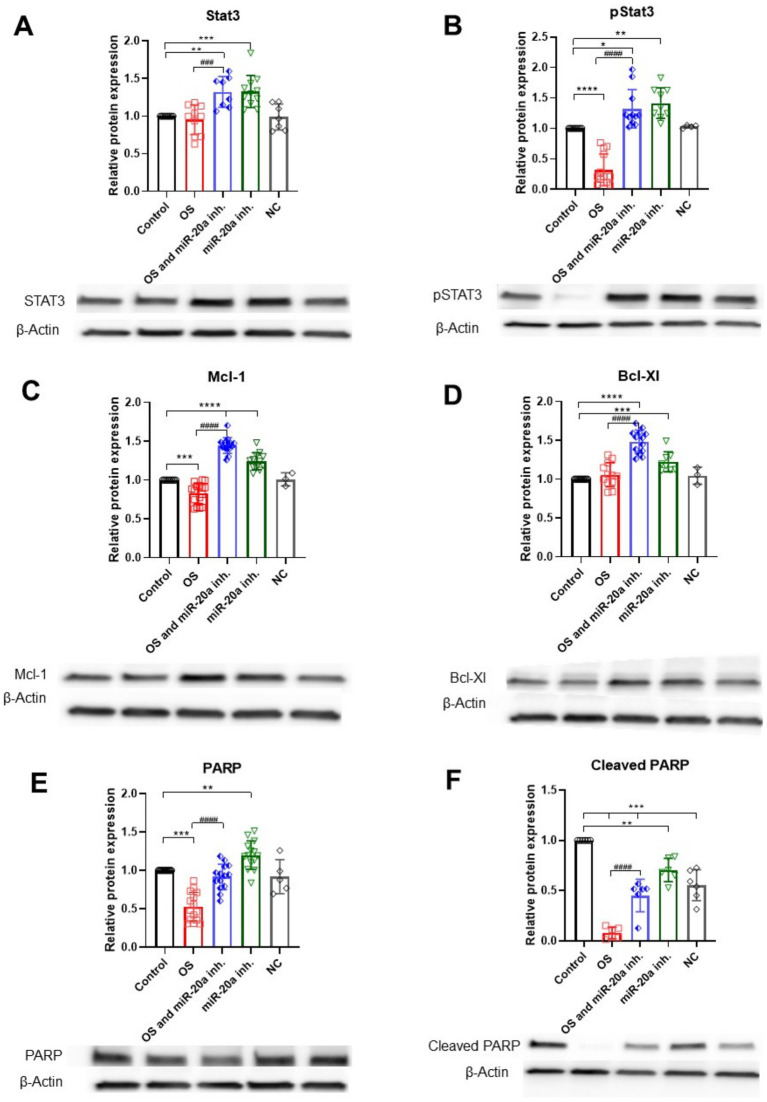
Anti-apoptotic protein expression under OS and miR-20a inhibition. The figure illustrates the effects of oxidative stress (H_2_O_2_) and miR-20a inhibition on the expression of key anti-apoptotic proteins, analyzed via Western blot. **(A)** STAT3: the groups miR-20a inhibition and OS, together with miR-20a inhibition, significantly increased STAT3 expression compared to the control. **(B)** pSTAT3: Phosphorylated STAT3 levels followed a similar trend, showing marked elevation with miR-20a inhibition during oxidative stress. **(C)** Mcl-1: miR-20a inhibition under OS conditions significantly upregulated Mcl-1 expression. **(D)** Bcl-XL: Bcl-XL levels increased under miR-20a inhibition and oxidative stress. **(E)** PARP: PARP expression was modulated under OS and miR-20a inhibition, indicating reduced apoptotic activity. **(F)** Cleaved PARP: miR-20a inhibition significantly reduced cleaved PARP levels, reflecting decreased apoptotic signaling. Statistical significance was determined using One-way ANOVA (*p < 0.05, **p < 0.01, ***p < 0.001, ****p < 0.0001; Stat3 *F* (4, 40) = 10.49; pStat3 *F* (4, 41) = 38.28; Mcl-1\F (4, 51) = 76.11; Bcl-Xl *F* (4, 44) = 30.55; PARP *F* (4, 58) = 34.25; Cleaved PARP *F* (4, 25) = 51.08).

**Figure 6:**
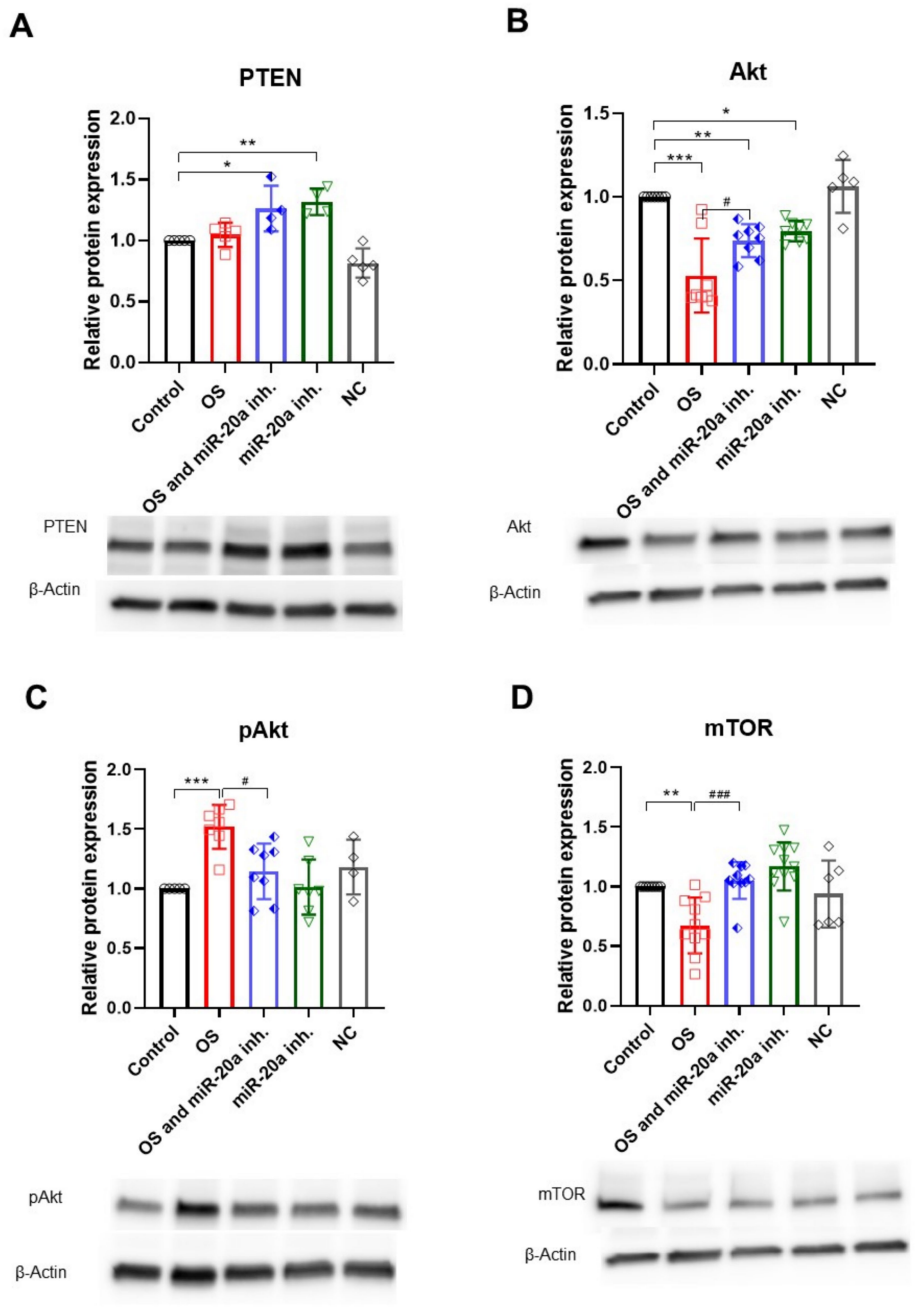
PI3K/Akt/mTOR pathway regulation under oxidative stress and miR-20a inhibition. The figure highlights the expression of proteins involved in the PI3K/Akt/mTOR signaling pathway. **(A)** H_2_O_2_ treatment did not significantly affect PTEN expression, whereas miR-20a inhibition led to an increase in PTEN levels under both oxidative stress (*p < 0.05) and non-oxidative stress (**p < 0.01) conditions. **(B)** Akt: Akt levels decreased after H_2_O_2_ treatment and partially recovered following miR-20a inhibition (**p < 0.01, ***p < 0.001). **(C)** pAkt: Phosphorylated Akt levels were reduced after H_2_O_2_ treatment but significantly increased with miR-20a inhibition (**p < 0.01, ***p < 0.001). **(D)** mTOR: mTOR expression decreased following H_2_O_2_ treatment (**p < 0.01) but was stabilized with miR-20a inhibition. Representative Western blot images and bar graphs highlight changes in protein expression across conditions. Statistical significance was determined using One-way ANOVA (*p < 0.05, **p < 0.01, ***p < 0.001; PTEN *F* (4, 18) = 14.12; Akt *F* (4, 31) = 17.93; *F* (4, 26) = 7.170; mTOR F (4, 40) = 9.193).

The negative control (NC) was used to determine whether the observed effects on protein expression were specific. Protein expression levels in the NC group did not show significant changes and were approximately the same as those in the control group (Figures 4–6). Another key player in apoptosis is caspase-3, often termed the “executioner” caspase due to its central role in cellular dismantling during apoptosis. Caspase-3 activation follows the release of cytochrome c and caspase-9 activation, leading to cleavage of pro-caspase-3. Increased caspase-3 expression indicates activation of the apoptotic pathway, with its levels significantly elevated (*p* < 0.01; F (4, 46) = 26.38) under OS (Figure 4C). Similar to Bax and cytochrome c, miR-20a inhibition under OS conditions significantly reduces caspase-3 levels (*p* < 0.001; F (4, 46) = 26.38), likely due to the downregulation of upstream pro-apoptotic proteins.

STAT3 is an important regulator of cell survival under OS, acting through the JAK/STAT3 signaling pathway. STAT3 is a target of miR-20a (Jee et al., 2012), so inhibiting this miRNA can upregulate STAT3, which in turn may promote cell survival. Active pSTAT3 induces the expression of pro-survival genes, such as Mcl-1 and Bcl-XL. Western blot analysis shows that miR-20a inhibition significantly upregulates STAT3 under both OS (*p* < 0.01; *F* (4, 40) = 10.49) and non-OS (p < 0.001; F (4, 40) = 10.49) conditions, with a corresponding increase in active pSTAT3, underscoring STAT3’s role in cell survival under OS (Figures 5A,B).

Under OS, the anti-apoptotic protein Mcl-1 is reduced (*p* < 0.001; *F* (4, 51) = 76.11), while Bcl-XL levels remain largely unchanged. Mcl-1 is also targeted by miR-20a (Liu et al., 2015), and our findings indicate that miR-20a inhibition significantly upregulates Mcl-1 (*p* < 0.0001; F (4, 51) = 76.11) under both OS and non-OS conditions, with even higher levels under OS. Bcl-XL levels also significantly increase (*p* < 0.0001; *F* (4, 44) = 30.55) in the OS with miR-20a inhibitor group, though miR-20a inhibition alone has minimal impact on Bcl-XL. This may suggest that miR-20a influences Bcl-XL expression indirectly, possibly through upstream regulation by STAT3 (Figures 5C,D).

PARP-1, a crucial component in the identification and restoration of DNA damage, shows reduced levels (*p* < 0.01; *F* (4, 58) = 34.25) under OS compared to the control group. However, in the H_2_O_2_ together with miR-20a inhibitor group, PARP-1 levels are upregulated. The group treated only with miR-20a inhibitor also shows a significant increase in PARP-1 levels (*p* < 0.01; F (4, 58) = 34.25) (Figures 5E,F), suggesting that miR-20a downregulation may enhance PARP-1, potentially promoting DNA repair in response to OS.

Inhibiting miR-20a under OS may support cell viability during early apoptosis, evidenced by reduced levels of pro-apoptotic proteins (Bax, cytochrome c, and caspase-3) and increased anti-apoptotic proteins (STAT3, PARP-1, Mcl-1 and Bcl-XL).

### 3.4 Inhibition of miR-20a regulates the PI3K/Akt/mTOR pathway by enhancing PTEN expression

The PI3K/Akt/mTOR pathway is a critical signaling cascade that regulates cell growth, proliferation, metabolism, and survival, playing a dual role in promoting cell survival or facilitating apoptosis under specific conditions, making it essential for cellular homeostasis. One of the key players in this pathway is PTEN (phosphatase and tensin homolog), which acts as a tumor suppressor and negatively regulates the PI3K/Akt pathway by dephosphorylating PIP3 back to PIP2, thus inhibiting Akt activation. MiR-20a targets PTEN, thereby affecting downstream molecules (Gao et al., 2019; Gong et al., 2022). Here we demonstrated that inhibition of miR-20a leads to increased PTEN expression in both groups treated with the inhibitor (*p* < 0.05 and *p* < 0.01; *F* (4, 18) = 14.12), suggesting that miR-20a might modulate PTEN expression in response to OS (Figure 6A).

The upregulation of PTEN results in reduced Akt (serine/threonine kinase) expression (*p* < 0.01; *F* (4, 31) = 17.93) in both groups with miR-20a inhibition, though this does not necessarily mean that Akt is a direct target of miR-20a; rather, it suggests a response to PTEN upregulation. In H_2_O_2_-treated cells, Akt level is reduced, while the combination of H_2_O_2_ and miR-20a inhibitor shows a partial restoration of Akt expression. Interestingly, the level of pAkt, essential for Akt activation and a marker of pathway activation, significantly increases (*p* < 0.001; *F* (4, 26) = 7.170) compared to the control and its inactive form (Akt) (Figures 6B,C), likely reflecting a cellular response to OS.

In the OS group treated with H_2_O_2_, mTOR levels significantly decreased (*p* < 0.01; *F* (4, 40) = 9.193) compared to the untreated control group. However, the introduction of a miR-20a inhibitor in the OS and miR-20a inhibitor group significantly restored mTOR expression levels (*p* < 0.001 (F (4, 40) = 9.193) compared to OS alone), nearly reaching those observed in the control group. Cells treated solely with the miR-20a inhibitor exhibited mTOR levels comparable to the control, suggesting no adverse effects of the inhibitor under non-stressed conditions (Figure 6D).

mTOR expression does not show significant changes across different conditions, implying that miR-20a inhibition and OS might not greatly impact total mTOR protein levels in this model (Figure 5D).

These results suggest that miR-20a inhibition modulates the PI3K/Akt/mTOR signaling pathway by upregulating PTEN and restoring Akt and mTOR activity under oxidative stress conditions. This regulatory effect highlights the potential of miR-20a inhibition to support neuronal cell survival and recovery by stabilizing key survival pathways disrupted by oxidative stress.

## 4 Discussion

MicroRNA-20a has emerged as an important regulator in the pathophysiology of SCI. Following SCI, miR-20a expression is significantly upregulated and remains elevated for at least a week post-injury (Liu et al., 2009; Strickland et al., 2011). This prolonged increase in miR-20a expression has been linked to secondary injury mechanisms that worsen the initial trauma. For instance, studies by Jee et al. (2012) demonstrated that exogenous administration of miR-20a into the spinal cord of adult mice led to increased neural apoptosis, suggesting that high levels of miR-20a may exacerbate neural cell death after SCI. Numerous genes are known to be mechanistically targeted by miR-20a, including neurogenin 1 (Ngn1), which is necessary for neuronal survival and differentiation (Bertrand et al., 2002). Furthermore, the infusion of an antisense miR-20a sequence, designed to inhibit miR-20a activity, was shown to reactivate the STAT3/Jak2/ERK1/2 pathways, increase PI3K/Akt phosphorylation, and downregulate apoptotic signals such as Bax and cytochrome c (Jee et al., 2012). These data suggest that miR-20a inhibition may contribute to cell survival and reduce apoptosis by modulating these pathways.

Oxidative stress is a fundamental component of secondary injury processes following SCI and is a key factor in neurodegeneration (Islam, 2017; Wang and Michaelis, 2010). After SCI, the transient hypoxia and subsequent reoxygenation lead to an excessive generation of ROS, which, when uncontrolled, can result in lipid peroxidation, mitochondrial damage, and DNA oxidation (Yu et al., 2023). ROS overproduction overwhelms antioxidant defenses and triggers oxidative stress reactions, exacerbating cellular damage and inflammation (Silva et al., 2014). In our study, we investigated the effects of OS and miR-20a inhibition on the induced pluripotent neural stem cells, assessing whether miR-20a inhibition could mitigate ROS-induced damage.

SCI-induced OS initiates a cascade that activates microglia, infiltrates leukocytes, and releases pro-inflammatory cytokines, thereby worsening injury through necrosis and apoptosis (Silva et al., 2014). Within 24 h post-SCI, apoptotic events predominate, with glial cells showing apoptosis peaks at multiple stages post-injury. This cascade is especially detrimental as mitochondrial dysfunction plays a central role; ROS generated by mitochondria cause further lipid peroxidation, ion imbalance, and mitochondrial membrane permeability, ultimately leading to a decline in ATP production and exacerbating neuronal death (Anjum et al., 2020).

Our findings demonstrate that H_2_O_2_-induced oxidative stress significantly reduced cellular metabolic activity during the early phases of exposure, as measured at 2- and 24- h post-treatment using the Alamar Blue assay. The consistent reduction in metabolic activity observed in the H_₂_O_₂_-only group underscores the early and sustained detrimental effects of oxidative stress on cellular function. However, treatment with the miR-20a inhibitor markedly mitigated this metabolic decline, particularly from the 24-h time point onward. This functional improvement suggests that miR-20a inhibition not only counteracts the damaging effects of ROS but may also play a reparative role by enhancing cellular resilience and promoting recovery mechanisms. Over time, the protective effects of the inhibitor became more pronounced, with metabolic activity approaching control levels, highlighting its potential to preserve cell viability under oxidative stress conditions. Importantly, miR-20a inhibition in cells not exposed to oxidative stress did not alter metabolic activity relative to controls, indicating that the inhibitor is non-toxic and does not adversely affect cell viability under baseline conditions.

The group treated with both H_2_O_2_ and the miR-20a inhibitor nearly restored metabolic function to control levels, suggesting that prolonged inhibition of miR-20a facilitates cellular recovery following oxidative stress. In contrast, the H_2_O_2_-only treated group showed sustained suppression of metabolic activity. The miR-20a inhibitor alone did not induce significant changes in metabolic activity compared to the control, indicating that its effects are primarily exerted in response to oxidative damage rather than through altering basal metabolic function.

Overall, our data demonstrate that miR-20a inhibition plays a critical role in mitigating the detrimental effects of oxidative stress on neural stem cells. While H_2_O_2_-induced oxidative stress led to a significant reduction in cellular metabolic activity, application of the miR-20a inhibitor promoted functional recovery. This protective effect became evident as early as 24 h post-treatment and continued to improve over time. By the 72-h mark, cells treated with both H_2_O_2_ and the miR-20a inhibitor exhibited near-normal levels of metabolic activity. These findings suggest that miR-20a is involved in the regulation of cellular responses to oxidative damage and that its inhibition may enhance the resilience of neural stem cells under oxidative stress. This highlights the potential therapeutic value of miR-20a inhibition in conditions characterized by oxidative damage, such as neurodegenerative diseases and spinal cord injuries.

OS also plays a dual role by regulating cell signaling pathways and influencing processes such as synaptic plasticity and neuronal survival (Gutierrez et al., 2006). ROS and RNS can act as secondary messengers in many CNS processes, with evidence that neurons can utilize ROS/RNS for intracellular responses to developmental and environmental cues (Kamsler and Segal, 2007; Serrano and Klann, 2004). At physiological levels, ROS support crucial cellular functions by regulating intrinsic signaling pathways, contributing to neural development, differentiation, polarization, synapse maturation, neurotransmission, and plasticity (Biswas et al., 2022). In adult neural stem cells, such as those in the hippocampus and retina, higher ROS levels are characteristic of quiescent NSCs compared to progenitors and differentiated neurons. These NSCs express specific antioxidant genes, suggesting a robust antioxidant defense that balances ROS signaling and prevents oxidative damage. This redox state is important for maintaining stemness and regulating the transition from quiescence to proliferation (Adusumilli et al., 2021; Mormone et al., 2023). However, when ROS levels exceed cellular buffering capacity, the CNS’s high oxygen demand and polyunsaturated lipid content leave it vulnerable to ROS-induced damage (Magistretti and Allaman, 2015). In our model, inhibition of miR-20a in H2O2-treated cells led to a progressive improvement in metabolic activity, suggesting that miR-20a may play a role in modulating the redox balance, potentially by influencing pathways that repair or compensate for ROS-induced damage.

Apoptosis is a critical component of neuronal response to OS, with both apoptosis and necrosis occurring within 24 h after SCI, and apoptosis predominating after this period (Yu et al., 2023). Following SCI, there is a biphasic peak in glial cell apoptosis, with the first peak at 24 h involving apoptosis spreading from the injury center to surrounding areas, and the second peak (7–10 days) primarily affecting oligodendrocytes, which comprise a large portion of apoptotic cells post-SCI. This extensive apoptotic response exacerbates CNS damage and impaired recovery (Yu et al., 2023).

Mitochondria, the primary source of ROS in cells, play a crucial role in OS-induced neuronal apoptosis. Under physiological conditions, mitochondrial functions such as cytosolic calcium buffering are tightly regulated. However, excessive ROS production compromises mitochondrial membrane integrity, increasing its permeability and leading to the release of cytochrome c and other pro-apoptotic factors, thereby initiating apoptosis (Anjum et al., 2020).

Consistent with this mechanism, our findings show that H_2_O_2_-treated cells exhibit elevated levels of pro-apoptotic proteins, including Bax and cytoplasmic cytochrome c, both indicators of mitochondrial dysfunction. Notably, inhibition of miR-20a under these conditions significantly reduced the expression of Bax and the release of cytochrome c, suggesting that miR-20a may play a protective role by modulating key apoptotic pathways and preserving mitochondrial integrity.

In the context of SCI, the intrinsic (mitochondrial) apoptotic pathway is a major mechanism of cell death, triggered by stressors such as ROS accumulation, DNA damage, or energy depletion. Pro-apoptotic members of the Bcl-2 family, including Bax and Bak, facilitate mitochondrial outer membrane permeabilization, which results in cytochrome c release. This release initiates the apoptotic cascade through activation of the initiator caspase-9, which subsequently activates caspase-3, the key effector responsible for executing apoptosis (Taylor et al., 2008).

Our results further demonstrate that miR-20a inhibition in H_2_O_2_-treated cells significantly reduces caspase-3 levels. While inactive caspase-3 is typically present in healthy cells, its upregulation during OS is likely driven by elevated levels of Bax and cytochrome c. The observed decrease in caspase-3 following miR-20a inhibition underscores its potential role in attenuating the apoptotic response induced by OS, thereby supporting neuronal cell viability.

In addition to mitochondrial damage, OS induces extensive neuronal injury through mechanisms such as lipid peroxidation, DNA damage, and membrane disruption, all of which impair cellular function and survival (Catalá and Díaz, 2016; Magistretti and Allaman, 2015). Poly PARP-1 is another critical player in OS response, facilitating DNA repair in the nucleus. However, excessive PARP-1 activation can lead to NAD^+^ depletion, ATP loss, and further ROS generation, amplifying mitochondrial dysfunction and apoptosis (Andrabi et al., 2006). In our findings, PARP-1 expression was significantly reduced in OS-only conditions, while PARP-1 upregulation with miR-20a inhibition suggests enhanced DNA repair activity, potentially counteracting OS-induced DNA damage.

Interaction of miR-20a with STAT3 highlights its role as a key regulatory molecule in SCI. STAT3 is a target of miR-20a and is crucial in SCI for promoting cell survival, astrogliosis, and glial scar formation (Carraro et al., 2009; Herrmann et al., 2008). When activated, STAT3 promotes the expression of anti-apoptotic proteins, such as Bcl-xL, Bcl-2, and Mcl-1, which are important for maintaining cell integrity and resisting apoptotic signals under stress (Epling-Burnette et al., 2001). In our study, we investigated the effects of miR-20a inhibition on STAT3 in the context of OS and found that miR-20a suppression significantly increased STAT3 expression in both OS and non-OS conditions. This upregulation of STAT3 was accompanied by an increase in pSTAT3, the active form of STAT3, suggesting that miR-20a inhibition enhances STAT3-mediated survival signaling. Enhancing STAT3 promotes the upregulation of survival-promoting genes, such as Mcl-1 and Bcl-XL. Consistent with this pathway, our results show that miR-20a inhibition significantly upregulated Mcl-1 and Bcl-XL in both OS and non-OS conditions, with Bcl-XL showing particular upregulation under OS. This increase in anti-apoptotic proteins likely contributes to enhanced cell survival under stress conditions, as these proteins counterbalance Bax, thereby preventing mitochondrial permeabilization and cytochrome c release.

In our study, the miR-20a inhibitor in OS conditions significantly reduced pro-apoptotic markers and increased anti-apoptotic proteins, indicating that miR-20a downregulation may counteract mitochondrial-mediated apoptosis, particularly by modulating Bcl-2 family members.

The PI3K/Akt signaling pathway is essential in the pathophysiology of SCI due to its regulatory role in neuronal apoptosis, survival, and cellular regeneration (Lv et al., 2024). As one of the key intracellular pathways, PI3K/Akt modulates various cellular functions by interacting with downstream targets, such as mTOR and FOXO1, to control processes like cell proliferation, apoptosis, autophagy, and metabolism (Wang et al., 2019). Multiple studies have demonstrated that PI3K/Akt activation helps to counteract OS, inflammation, and apoptosis following SCI, supporting nerve regeneration and functional recovery by improving the cellular environment (Goswami et al., 1999; Yang et al., 2021; Zhao et al., 2022).

In SCI, neuronal apoptosis is a primary contributor to spinal cord dysfunction and a significant obstacle to recovery efforts. Inhibition of apoptosis has shown promise in improving SCI outcomes (Varma et al., 2013). Activation of the PI3K/Akt pathway is known to decrease the expression of pro-apoptotic markers, such as caspase-3 and caspase-9, while inhibiting this pathway can lead to cell cycle arrest and enhanced apoptosis (Shin et al., 2012; Yinming et al., 2018). Akt activation reduces apoptosis by phosphorylating and inactivating pro-apoptotic proteins, such as Bax and caspase-9, thereby maintaining mitochondrial integrity and preventing the release of cytochrome c, a key step in apoptosis (Chan, 2004; Fresno Vara et al., 2004). Supporting this role, several studies have shown that activating PI3K/Akt after SCI can inhibit neuronal apoptosis, reduce OS, and prevent disruption of the blood-spinal barrier, ultimately slowing secondary injury progression (Li et al., 2019). Our study supports these findings by showing that miR-20a inhibition leads to the upregulation of PTEN. This tumor suppressor negatively regulates the PI3K/Akt pathway, playing a critical role in cellular signaling and survival. PTEN functions by dephosphorylating PIP3 to PIP2, thus inhibiting Akt activation (Lv et al., 2024). In our findings, inhibition of miR-20a led to increased PTEN expression, which corresponded with a reduction in total Akt levels, particularly in OS conditions. Interestingly, while total Akt levels decreased, we observed a significant increase in pAkt levels under OS, suggesting an adaptive response to stress. This upregulation of pAkt indicates a compensatory mechanism that may help to maintain cell survival and counteract apoptosis despite increased PTEN expression.

The dual regulatory role of PI3K/Akt in SCI recovery extends to anti-apoptotic mechanisms, where it balances anti-apoptotic proteins (e.g., Bcl-2 and Bcl-XL) with pro-apoptotic proteins (Chan et al., 2010). For instance, treadmill exercise has been shown to activate PI3K/Akt in injured spinal cords, leading to an increased Bcl-2/Bax ratio and enhanced neurotrophic factor expression, all of which contribute to cell survival and motor recovery (Jung et al., 2014). Despite the significant modulation of Akt and STAT3 pathways, we observed that total mTOR levels displayed variable trends across the experimental groups. Notably, OS reduced mTOR expression, but miR-20a inhibition effectively restored mTOR levels, suggesting its role in counteracting OS-induced suppression of mTOR signaling. This restoration aligns with the observed protective effects, implying that miR-20a inhibition may support cellular recovery through mTOR-dependent mechanisms alongside PI3K/Akt and STAT3 pathways. This highlights the complexity of the molecular interplay in oxidative stress and miR-20a modulation.

Our study demonstrates that miR-20a plays a critical role in modulating oxidative stress responses and apoptosis in neural stem cells, particularly under conditions mimicking spinal cord injury. Inhibition of miR-20a not only reduced pro-apoptotic markers and mitochondrial dysfunction but also enhanced cellular survival by upregulating protective pathways, including STAT3 and PI3K/Akt/mTOR. These findings suggest that targeting miR-20a may represent a promising therapeutic strategy for mitigating secondary injury and promoting neuroprotection following spinal cord injury.

## 5 Conclusion

In summary, our findings indicate that miR-20a plays a critical role in OS through its regulation of STAT3 and apoptotic pathways. By inhibiting miR-20a, STAT3 is upregulated, which enhances the expression of key anti-apoptotic proteins such as Mcl-1 and Bcl-XL, thus supporting NSCs survival. This suggests that miR-20a is a promising therapeutic target for managing SCI by modulating apoptosis and promoting cellular resilience in response to oxidative and inflammatory stressors.

Given the complex and often damaging role of oxidative stress in SCI, miR-20a inhibition may have therapeutic value by boosting resistance against ROS-induced cellular damage, especially relevant in neurodegenerative conditions where oxidative stress is prominent. Our data show that miR-20a inhibition modulates PTEN and STAT3 within the PI3K/Akt pathway, activating anti-apoptotic signaling and enhancing cell survival. The observed regulatory effects of miR-20a inhibition on STAT3 and PI3K/Akt pathway components underscore its therapeutic potential in protecting neurons from OS and apoptosis.

Collectively, these findings support miR-20a inhibition as a promising approach to counteract oxidative damage and enhance neuronal adaptability in SCI. Future studies should explore *in vivo* applications of miR-20a modulation and investigate its interactions within additional apoptotic pathways to deepen our understanding of its role in OS response and its therapeutic potential in SCI.

## Data Availability

The original contributions presented in the study are included in the article/supplementary material, further inquiries can be directed to the corresponding author.

## Author’s note

The iPSC-NP cell line used in this study was originally obtained from Brigitte Onteniente as part of the European project STEMS. It is now commercially available as PCi-NPC from Phenocell (Grasse, France).

## Supplementary material

SUPPLEMENTARY FIGURE 1 Immunostaining of human induced stem cells derived neural precursors confirm their specific properties. Derived cells were positive for GFAP **(A)**, NF-L **(B)**, Sox2 **(C)**, MAP2 **(D)**, Nestin **(E)** and Pax 6 **(F)**. Scale bar 20 um.

SUPPLEMENTARY FIGURE 2 Flow cytometry analysis of apoptotic cell populations under oxidative stress conditions. Flow cytometry plots show the distribution of live, early apoptotic, late apoptotic, and necrotic cells for: **(A)** Control, **(B)** 100 μM H_2_O_2_, **(C)** 200 μM H_2_O_2_, and **(D)** 500 μM H_2_O_2_. In the Control group **(A)**, the majority of cells are viable (89.6%), with only a small proportion in early (6.91%) or late apoptosis (2.70%), reflecting minimal baseline stress. Treatment with 100 μM H_2_O_2_ **(B)** reduces the live cell population to 60.3%, with a marked increase in early apoptosis (24.2%) and late apoptosis (11.1%). At 200 μM H_2_O_2_ **(C)**, cell viability declines further (52.7%), accompanied by an increase in early apoptosis (39.1%) and a modest rise in late apoptosis (6.59%). Exposure to 500 μM H_2_O_2_ **(D)** results in the most pronounced effect, with live cells dropping to 38.2%, early apoptosis decreasing to 3.70%, and a substantial increase in late apoptosis (30.8%), reflecting severe oxidative damage. These results demonstrate a dose-dependent cytotoxic effect of H_2_O_2_, with increasing concentrations leading to reduced cell viability and a transition from early to late apoptosis, indicative of advanced cellular damage under oxidative stress.
