# Correction: Inhibition of miR-20a promotes neural stem cell survival under oxidative stress conditions

**DOI:** 10.3389/fnins.2025.1655293

**Published:** 2025-07-17

**Authors:** Frontiers Production Office

**Affiliations:** Frontiers Media SA, Lausanne, Switzerland

**Keywords:** microRNA-20a, neural stem cells, oxidative stress, neural apoptosis, neuroprotection

There was a mistake in Table 1 as published. Commas and spaces were erroneously removed from the third column. In rows 5 and 6, “7986” should be “79, 86”. In row 12, “351917” should be “35, 19, 17”. The corrected Table 1 appears below.

**Table 1:** The list of the primary antibodies.

**Antibodies name**	**Catalog number#**	**Molecular weight (kDa)**	**Isotope**	**Dilution solution**	**Dilution**
mTOR	2983	289	Rabbit mAb	BSA	1:1,000
Akt	9272	60	Rabbit mAb	BSA	1:1,000
PTEN	9559	54	Rabbit mAb	BSA	1:1,000
Phospho-Akt	4060	60	Rabbit mAb	BSA	1:1,000
Stat3	9139	79, 86	Mouse mAb	Milk	1:1,000
Pospho-Stat3	9145	79, 86	Rabbit mAb	BSA	1:2,000
PARP	9542	116	Rabbit mAb	BSA	1:1,000
Cleaved PARP	9541	89	Rabbit mAb	BSA	1:1,000
Mcl-1	94296	40	Rabbit mAb	BSA	1:1,000
Bcl-XL	2764	30	Rabbit mAb	BSA	1:1,000
Bax	14796	20	Rabbit mAb	BSA	1:1,000
Caspase-3	14220	35, 19, 17	Rabbit mAb	BSA	1:1,000
Cytochrome c	11940	14	Rabbit mAb	BSA	1:1,000
β-Actin	A2228	42	Mouse mAb	TBST	1:2,000

The original version of this article has been updated.

