# Supplementary figures and images for: Inhibition of miR-20a promotes neural stem cell survival under oxidative stress conditions
Source: Front Neurosci. 2025 Jun 26;19:1601101. doi: 10.3389/fnins.2025.1601101 (PMC12240997; doi:10.3389/fnins.2025.1601101)

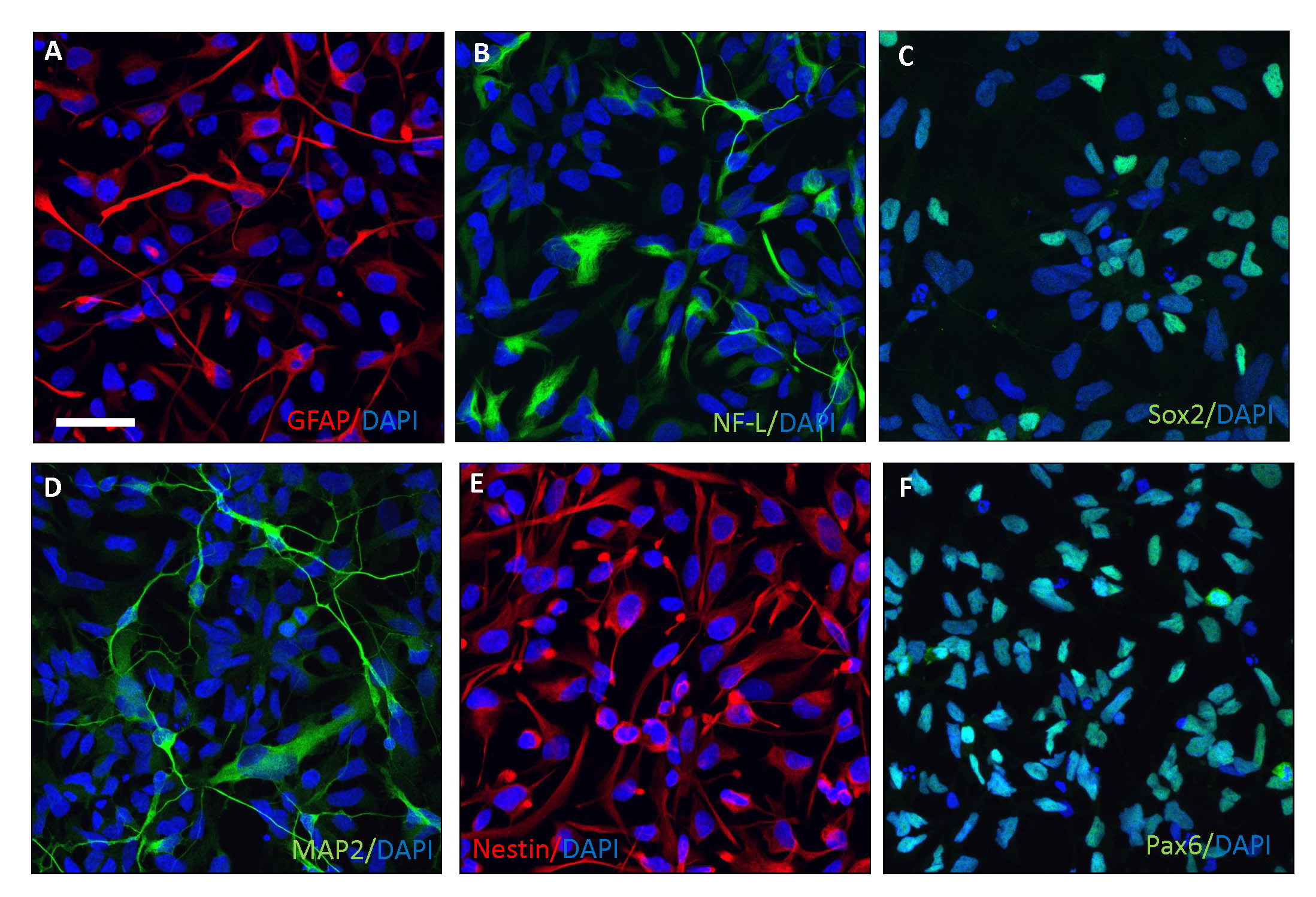

Supplement: SUPPLEMENTARY FIGURE 1 — Immunostaining of human induced stem cells derived neural precursors confirm their specific properties. Derived cells were positive for GFAP (A), NF-L (B), Sox2 (C), MAP2 (D), Nestin (E) and Pax 6 (F). Scale bar 20 um. [file Image_1.JPEG]

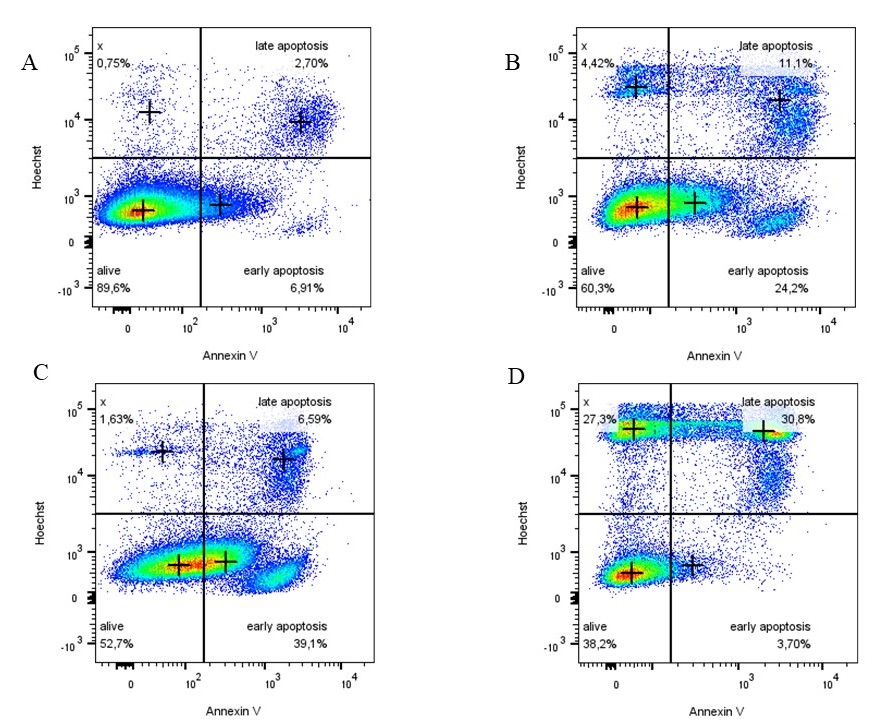

Supplement: SUPPLEMENTARY FIGURE 2 — Flow cytometry analysis of apoptotic cell populations under oxidative stress conditions. Flow cytometry plots show the distribution of live, early apoptotic, late apoptotic, and necrotic cells for: (A) Control, (B) 100 μM H2O2, (C) 200 μM H2O2, and (D) 500 μM H2O2. In the Control group (A), the majority of cells are viable (89.6%), with only a small proportion in early (6.91%) or late apoptosis (2.70%), reflecting minimal baseline stress. Treatment with 100 μM H2O2 (B) reduces the live cell population to 60.3%, with a marked increase in early apoptosis (24.2%) and late apoptosis (11.1%). At 200 μM H2O2 (C), cell viability declines further (52.7%), accompanied by an increase in early apoptosis (39.1%) and a modest rise in late apoptosis (6.59%). Exposure to 500 μM H2O2 (D) results in the most pronounced effect, with live cells dropping to 38.2%, early apoptosis decreasing to 3.70%, and a substantial increase in late apoptosis (30.8%), reflecting severe oxidative damage. These results demonstrate a dose-dependent cytotoxic effect of H2O2, with increasing concentrations leading to reduced cell viability and a transition from early to late apoptosis, indicative of advanced cellular damage under oxidative stress. [file Image_2.JPEG]
